# Pharmacological and non-pharmacological modulation of endoplasmic reticulum stress in pediatric diabetes

**DOI:** 10.3389/fendo.2025.1690478

**Published:** 2025-10-29

**Authors:** Aristeidis P. Giannakopoulos

**Affiliations:** Division of Pediatric Endocrinology, Department of Pediatrics, University Hospital of Patras, Patras, Greece

**Keywords:** pediatric diabetes, endoplasmic reticulum stress, unfolded protein response, exercise, metformin

## Abstract

In all forms of pediatric diabetes, the endoplasmic reticulum (ER) stress acquires a significant role, as a factor that contributes to the dysfunction and apoptosis of the pancreatic β- cells. The integrity of the ER response is critical as a molecular mechanism for alleviating stress during insulin biosynthesis and processing, regardless of the diabetes subtype. While achieving euglycemia remains central to diabetes management, there is growing recognition that targeting ER stress presents a promising therapeutic strategy, given that accumulating evidence shows that ER stress acts not only as a consequence but also as a key contributor to diabetes pathogenesis. This review explores the mechanisms of ER stress across all forms of diabetes, discusses both pharmacological and non-pharmacological approaches to modulating ER stress—with particular attention to medications that are already approved for use in children, such as metformin —and examines the potential of combining ER stress modulation with insulin therapy in order to optimize the metabolic homeostasis for the β-cell function and survival.

## Introduction

Childhood Type 1 Diabetes (T1D) represents a growing global health challenge, with epidemiological data indicating a sustained increase in incidence among children and adolescents in the last decades from 9.31 to 11.61/100 000 population and is currently the leading cause of macro-vascular and micro-vascular disease including kidney, heart, and eye (1). Technological advances in T1D such as the closed-loop insulin delivery systems have improved glycemic control and reduced the burden of the disease improving patients’ quality of life (2). Type 2 diabetes (T2D), which is the most diagnosed form in adults, accounting for over 90% of all diabetes diagnosed cases has also increased in the adolescent population due to obesity epidemic in the last decades (3). Apart from T1D and T2D, which are the main polygenic forms caused by distinct underlying mechanisms, some children develop diabetes due to single-gene mutations (monogenic diabetes). These genetic variants affect insulin secretion. Monogenic diabetes is classified into two groups: maturity-onset diabetes of the young (MODY) and neonatal or early-onset diabetes mellitus.

**Figure 1:**
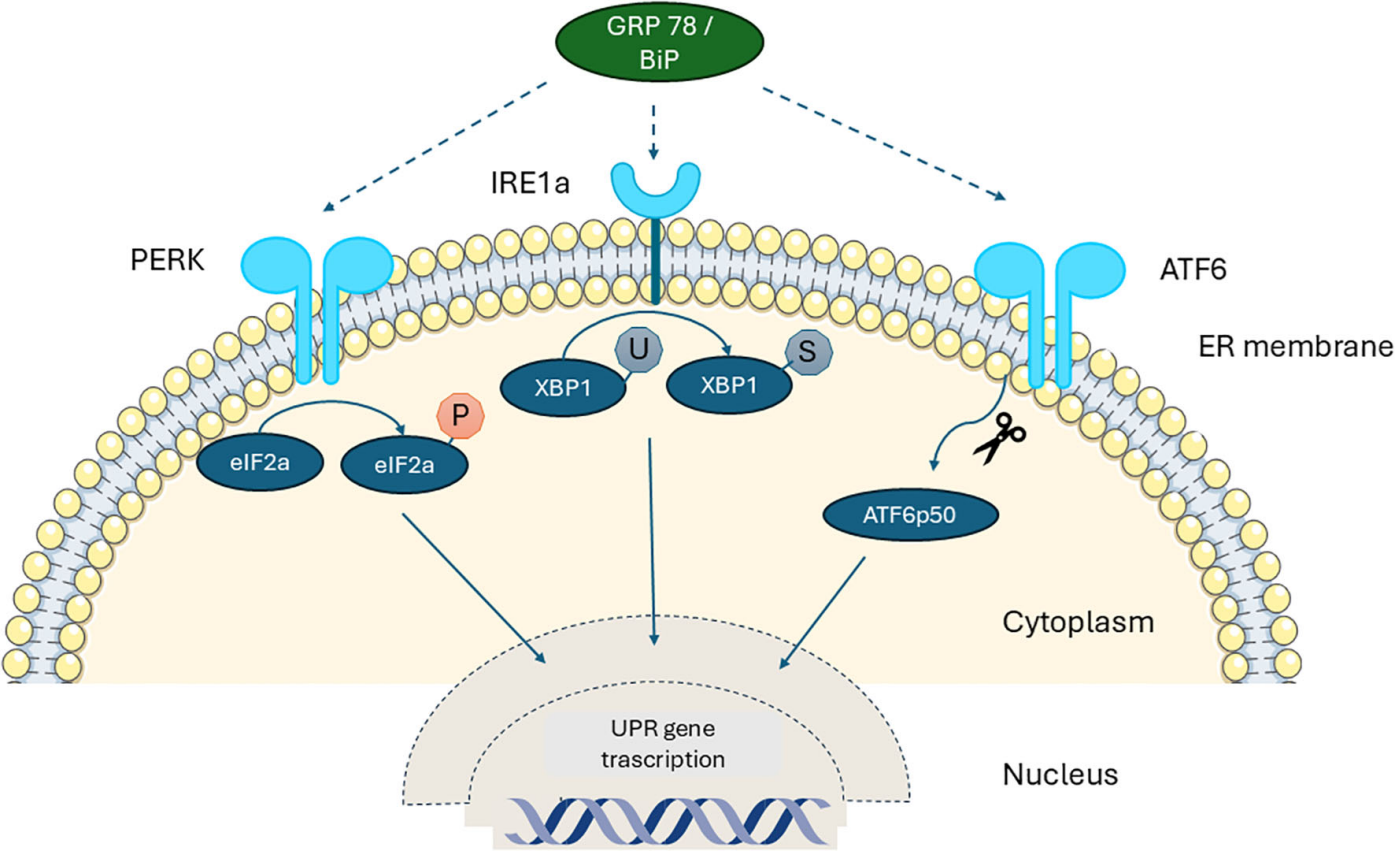
Schematic diagram of the unfolded protein response (UPR) in the β-cell. Under normal conditions without stress, the three UPR sensors— IRE1a, ATF6, and PERK—remain inactive as monomers bound to the ER chaperone GRP78/BiP. When misfolded proinsulin accumulates beyond the ER’s clearance capacity, BiP detaches from these sensors and binds the exposed hydrophobic regions of the misfolded proteins, releasing IRE1, ATF6, and PERK. This release activates downstream signaling pathways and UPR target genes, which work to restore ER homeostasis by enhancing molecular chaperones and reducing retention of misfolded proinsulin. PERK: Protein Kinase R-like ER, IRE1a: Serine–threonine Protein Kinase–Endoribonuclease XBP-1: X-box Binding Protein-1, GRP78/BiP: 78 kDa Glucose-Regulated Protein/Binding immunoglobulin Protein.

In recent years substantial evidence shows that all forms of pediatric diabetes, despite having different underlying causes, share a common feature: the presence of ER stress in β-cells. At the cellular level, ER is the key organelle that links energy management and protein secretion. ER is classified morphologically into two distinct types: rough and smooth ER. This structural difference corresponds to their specialized functions – smooth ER synthesizes phospholipids and cholesterol, whereas the rough ER is primarily involved in the synthesis and export of proteins and glycoproteins. Overall, the ER plays a vital role in protein synthesis, modification, and trafficking. Additionally, it serves as a crucial site for the mobilization and regulation of calcium release (4, 5). This organelle is also involved in the oxidation-reduction and autophagy molecular pathways (6). In the ER lumen, proteins through post-translational modifications, such as glycosylation, hydroxylation and disulfide bond formation, acquire their 3-dimensional structure and their final biological properties (7). The homeostatic regulation of this process is essential for proper protein folding and cell survival, especially for cell populations that have intense secretory function such as hepatocytes and pancreatic β-cells (8, 9). In the case of β-cells, over 50% of their total mRNA is dedicated to proinsulin synthesis (10) and therefore depend heavily on ER to meet the increased demand for insulin biosynthesis and secretion in response to elevated nutrient levels, ensuring glucose homeostasis in the body. Indeed, the first studies had shown that islet cells from individuals with T1D displayed ER stress response with demonstration of activation of specific ER stress related pathways (11, 12). The dependence of β-cells on ER function renders them susceptible to ER stress during increased insulin demands, requiring proper pro-insulin folding. Impaired ER homeostasis, caused by the imbalance between protein-folding capacity and protein-folding load, induces ER stress (13), mediated by a series of receptors and signaling pathways that monitor protein synthesis. The adaptive reaction developed to ER stress is the Unfolded Protein Response (UPR), which aims to decrease the synthesis of new proteins while increasing the functional capacity of the ER (14). Serine–threonine protein kinase–endoribonuclease IRE1α, protein kinase R-like ER kinase (PERK, also known as EIF2AK3) and the cAMP-dependent transcription factor ATF6 are the three ER proteins involved in the UPR. The molecular chaperone glucose regulated protein 78/Binding immunoglobulin Protein (GRP78/BiP) localized to the ER lumen is key to this process. Under homeostatic conditions, IRE1α, PERK and ATF6 are bound to and inhibited by the GRP78/BiP. When ER stress is initiated, GRP78/BiP disassociates from these molecules resulting in their activation. IRE1α is activated by auto-phosphorylation and subsequently phosphorylates other signaling proteins. It also splices the X-box binding protein 1 (XBP-1) mRNA leading to the generation of an alternatively spliced form of XBP-1, a transcription factor that induces the expression of other molecular chaperone proteins. In addition, GRP78/BiP stimulates PERK kinase activity, and this enzyme phosphorylates eukaryotic initiation factor 2α (eIF2α), thereby inhibiting the activity of this essential translational enhancer. Finally, activating transcription factor 6 (ATF6) is released from the ER/Golgi apparatus by proteolytic processing, whereby it translocates to the nucleus and enhances expression of other ER-associated proteins including GRP78/BiP (15). ER stress inhibits insulin synthesis and secretion via PERK-eIF2α. Several studies have investigated the role of PERK and eIF2α in pancreatic β-cells and their impact on insulin secretion. A mutation of PERK and eIF2α in β-cells can alter the disulfide bond structure of proinsulin, causing increased misfolding and inadequate insulin secretion (8). ER stress can also inhibit insulin secretion through the IRE1α-XBP1 pathway, activating CHOP which is a pro-apoptotic transcription factor linked to the ER stress response pathway (16, 17). Calcium facilitates the fusion of insulin precursor granules to the plasma membrane of pancreatic β -cells in the insulin secretion pathway and prolonged ER stress decreases ER calcium content, inhibiting insulin secretion (18, 19). When ER stress increases to uncompensated levels the cells undergo apoptosis (13). This highlights the critical role of the UPR in maintaining ER homeostasis and supporting normal β-cell function (**Figure 1**).

In all forms of diabetes, the genetic susceptibility to ER stress response acquires a significant role, as a factor that contributes to the dysfunction and apoptosis of the pancreatic β-cells. In both T1D and T2D as well in monogenic and other syndromic types of diabetes, the intactness of UPR is important as a molecular stress-alleviating mechanism in the production and post-modification process of the insulin molecule irrespective of the triggering factor (20, 21). This is also well substantiated in the case of Wolfram syndrome, where diabetes is a core element of its phenotype, caused by mutations in the *WFS1* or *CISD2* genes. Wolfram syndrome is considered a prototype ER disorder, as both WFS1 and CISD2 proteins localize to the ER and are crucial for maintaining ER homeostasis. WFS1 regulates the UPR via interaction with ATF6 and helps maintain ER and cellular calcium balance, essential for insulin folding and secretion (22). Pathogenic WFS1 variants can cause protein misfolding, triggering chronic ER stress that impairs β-cells, neurons, retinal ganglion cells, and oligodendrocytes, leading to tissue degeneration. The exact function of *CISD2* gene is still not clear, but it has been shown to play a role in the regulation of pro-apoptotic molecule calpain 2 (23). ER stress also contributes to mitochondrial dysfunction, making Wolfram syndrome a systemic disease (22, 24, 25).

Another autosomal recessive disorder in which diabetes is a cardinal feature, is Wallcott Rallison Syndrome, manifested early in infancy and caused by mutations in the *EIF2AK3* gene (26). This gene encodes PERK that, along with IRE1 and ATF6, senses ER stress and activates the UPR. When activated, PERK phosphorylates eIF2α, leading to reduced protein synthesis to alleviate ER stress.

Mutations in YIPF5, a protein involved in ER-to-Golgi trafficking, have been shown to cause neonatal diabetes and microcephaly due to increased ER stress in pancreatic β-cells. Loss of *YIPF5* function results in the retention of proinsulin within the ER, leading to pronounced ER stress and β-cell failure. Functional studies reveal that YIPF5 deficiency enhances β-cell sensitivity to ER stress-induced apoptosis, underscoring the critical role of proper ER homeostasis in β-cell survival and insulin secretion. De Franco et al. demonstrated that ER stress is a central pathogenic mechanism in YIPF5-related diabetes, highlighting the importance of protein trafficking pathways for both β-cell function and neurological development (27).

In addition, certain gene variants causing Maturity-Onset Diabetes of the Young (MODY) increase physiological ER stress in pancreatic β-cells, contributing to cell dysfunction and diabetes progression. The MODY subtypes involve mutations in genes such as *HNF4A* (MODY1), *GCK* (MODY2), *HNF1A* (MODY3), *PDX1* (MODY4), *PAX4* (MODY9), and *INS* (MODY10)—each with distinct but converging mechanisms that exacerbate ER stress (6). For example, HNF4A mutations can upregulate ANKS4b, which interacts with the ER chaperone GRP78/BiP, sensitizing β-cells to ER stress-induced apoptosis (28). Similarly, GCK mutations increase pro-apoptotic CHOP expression and impair insulin secretion under ER stress. Mice carrying a missense mutation in the *GCK* gene exhibited impaired β-cell function accompanied by elevated levels of CHOP expression in their islets (29). HNF1A mutant β-cells are characterized by diminished XBP1 and BiP, leading to heightened vulnerability to ER stress, and reduced insulin production (27, 30). PDX1 mutations hinder the expression of essential genes regulating ER homeostasis, while INS gene mutations can result in the accumulation of misfolded proinsulin that disrupts wild-type insulin folding and further aggravates ER stress (6). The above underscores not only the pathophysiological consequences of MODY mutations but also highlights ER stress as basic factor of β-cell loss as all these gene defects disrupt protein folding, impair adaptive unfolded protein response (UPR) signals, and render β-cells highly susceptible to functional decline and apoptosis (20).

## Genetic susceptibility factors for ER stress in diabetes

Gene variants associated with susceptibility to ER stress and the activation of the UPR may facilitate the disruption in ER homeostasis, which is important for efficient insulin production and secretion (6, 22). Such genes involved in ER stress regulation are *JAZF1* and *RNF213*. *JAZF1* (Juxtaposed with Another Zinc Finger Protein 1) participates in ribosome biogenesis and protein synthesis, and its reduced expression impairs protein folding capacity in the ER, leading to increased ER stress and metabolic dysfunction (31). Conversely, *RNF213* (Ring Finger Protein 213), encoding an E3 ubiquitin ligase, appears to function as a negative regulator of ER stress. Suppression of *RNF213* expression reduced ER stress levels and improves insulin sensitivity, suggesting a protective role against the development of diabetes (32). Emerging evidence also links *RNF213* to lipid metabolism and inflammatory responses, further supporting its role in metabolic homeostasis (33).

In the context of T2D it has been found that ER stress is increased by factors like sustained hyperglycemia and oxidative stress, contributing β-cell dysfunction and insulin resistance (34, 35). In this process, pathologic variants of a gene named *MAP3K5* disrupts its normal interaction with stress-responsive cis-regulatory elements (CREs), leading to increased ER stress and β-cell apoptosis (36).

## ER stress in T1D

In T1D, ER stress is a significant factor in β-cell dysfunction and disease progression. Autoimmune-mediated β-cell destruction leads to insulin deficiency, and the remaining β-cells experience increased workload due to compensatory insulin production. Under these conditions of high metabolic demand and underlying inflammation, misfolded and unfolded proteins accumulate leading to elevated ER stress (37). When the UPR fails to restore homeostasis, chronic ER stress activates pro-apoptotic pathways—particularly CHOP and JNK signaling—leading to β-cell apoptosis. This progressive loss of β-cell mass is a hallmark of both T1D and T2D (6, 22, 38). While improved glycemic control can alleviate ER stress to some extent, the presence of inflammation due to autoimmunity may continue to sustain elevated ER stress which impairs the remaining capacity of insulin biosynthesis and secretion (6). Importantly, in T1D, ER stress not only compromises β-cell function but also contributes to their immune-mediated destruction. Disruption of ER calcium homeostasis enhances the production of aberrant or neo-antigens, which are presented to the immune system, triggering an autoimmune response. In addition, β-cell exposure to cytokines, present in the islet environment during the different stages of insulitis, for example, type I interferons (mostly IFNα) at the early stages of inflammation, and then IFNγ plus IL-1β, tumor necrosis factor (TNF) and, potentially, IL-17 at the latest stages enhance the presentation of β-cell neoantigens to immune cells, facilitating T-cell–mediated β-cell targeting and accelerating β-cell loss (39–41). The above processes comprise a vicious circle of enhanced autoimmunity and β-cell vulnerability (39, 42).

## ER stress in T2D

In T2D, chronic hyperglycemia, lipotoxicity, and pro-inflammatory cytokines drive the accumulation of misfolded proinsulin and induce persistent ER stress in pancreatic β-cells (43). UPR initially strive to maintain insulin synthesis, but sustained ER stress impairs these responses, activating apoptotic signaling cascades including PERK–eIF2α–ATF4–CHOP, IRE1–JNK, and NF-κB, leading to β-cell apoptosis (44, 45). In line with this, studies have demonstrated that islets from patients with T2D exhibited elevated levels of CHOP (46) and pancreatic tissue from T2D individuals with obesity showed a sixfold increase in perinuclear CHOP expression compared to those from individuals with obesity but without T2D (47). ER stress further contributes to β-cell senescence, defined by a non-proliferative phenotype that secretes senescence-associated secretory phenotype (SASP) factors exacerbating dysfunction in insulin production and secretion (34). Of note is that recent studies have shown that defects in proinsulin/insulin processing and ER stress markers progressively worsen during the transition from normal glucose tolerance to impaired glucose tolerance, leading to T2D mellitus (47). Brusco et al. analyzed pancreas biopsies from metabolically characterized living donors analyzing ER stress and β-cell differentiation markers in patients with impaired glucose tolerance and T2D and they found that increased ER stress and altered insulin processing/secretion induce loss of β-cell phenotype across the distinct stages of T2D progression. The early loss of β-cell identity was linked to β-cell exhaustion and clinically overt diabetes (48). Hyperglycemia also intensifies oxidative stress via increased mitochondrial and NADPH oxidase-derived reactive oxygen species (ROS), overwhelming the inherently low antioxidant defenses of β-cells (49). ROS disrupt key transcription factors (such as PDX-1 and MafA), decrease ATP generation through UCP2 induction, and suppress insulin secretion (50). Oxidative stress and ER stress synergistically form a vicious cycle, as ROS reduces ER redox balance and promotes protein misfolding, which in turn further elevates ROS production, promoting the β-cell dysfunction (51).

Moreover, ER stress activates inflammatory signaling through NF-κB and JNK pathways, resulting in upregulation of pro-inflammatory cytokines including IL-1β and TNFα, and stimulation of TXNIP expression, which triggers NLRP3 inflammasome activation for further cytokine release (15, 52). This fuels a deleterious feedback loop, exacerbating ER stress and speeding β-cell apoptosis in T2D. The intricate interplay between hyperglycemia, oxidative stress, ER stress, and inflammation leads to progressive β-cell dysfunction and loss. A clinical trial with the name GLUCOSTRESS (NCT02368704) investigated how activation of ER stress pathways contribute to the pathophysiology of T2D, with a particular focus on insulin resistance and related metabolic complications but no results have yet been publicly available.

## Exercise and ER stress in T1D and T2D

Chronic ER stress is recognized as a central contributor to β-cell dysfunction and death in T1D and T2D. When the UPR cannot adapt to persistent stress—often caused by inflammation or autoimmunity—cellular apoptosis is triggered, and β-cell loss is accelerated (53). Certain proteins play key roles in how exercise alleviates diabetes-related ER stress, including AMPK, PPARδ, and PGC-1α, which are vital in suppressing ER stress during physical activity. AMPK (AMP-activated protein kinase), found broadly in eukaryotic cells, regulates cellular energy balance and metabolism. The nuclear receptor PPARδ reduces ER stress by controlling gene transcription, suppressing inflammatory factors, increasing antioxidant enzymes, and enhancing lipid metabolism (54). During exercise, AMPK and PPARδ act synergistically to suppress ER stress (55). In another study, Cheang et al. demonstrated that exercise activates AMPK and PPARδ in vascular endothelial cells of diabetic mice, lowering ER stress-related proteins p-eIF2α, XBP1s, and ATF6, which improves vascular function (56). Moreover, PGC-1α, an important transcriptional coactivator that regulates energy metabolism, mitochondrial biogenesis, and oxidative stress responses is activated via the AMPK-PGC-1α pathway, reducing ER stress in diabetic mice (57). Recent scientific evidence shows that exercise can induce the release of circulating factors—collectively known as exerkines—that have protective effects on pancreatic β-cells exposed to ER stress. For instance, human β-cell lines (EndoC-βH1) and isolated human islets exposed to serum from individuals following 8–12 weeks of high-intensity exercise showed marked resistance to ER stress-induced apoptosis. This protective effect was present even when serum collected from participants with T1D or T2D was used, indicating a direct muscle-to β-cell communication mediated by secreted factors (58, 59). The role of exercise in modulating ER stress is not limited to the pancreas. Exercise has been shown to alleviate ER stress also in metabolic tissues, particularly the liver and adipose tissue. In mice fed a high-fat diet, exercise reduced activation of the ER stress PERK-eIF2α-ATF4 signaling pathway, improved mitochondrial function, and decreased hepatic lipid accumulation (60). A recent study showed that high-intensity interval training (HIIT) could prevent metabolic dysfunction-associated steatotic liver disease in high-fat diet-fed mice by preserving insulin sensitivity, promoting beta-oxidation and preventing ER stress. Over 10 weeks, high intensity exercise of 12 minutes, 3 times weekly prevented weight gain, maintained insulin sensitivity and preserved ER and mitochondrial function. HIIT also showed anti-inflammatory and anti-lipogenic effects through the increased hepatic fibronectin type III domain containing 5/irisin (61). In adipose tissue ER stress impacts key adipogenic transcription factors such as proliferator-activated receptor γ (PPARγ) and CCAAT-enhancer-binding proteins (C/EBPs) along with their interaction with other signaling pathways and exercise may have an enhancing role through this direction (62). Exercise not only modulates systemic metabolic health but also activates cellular repair pathways, such as autophagy via AMPK/PGC-1α signaling (58).

Taken together, these data underscore the role of exercise in protecting β-cells and alleviating ER stress across multiple tissue types.

## Pharmacological modulation of ER stress in pediatric diabetes

Pharmacological modulation of ER stress represents a promising therapeutic approach for both T1D and T2D as well as the other forms of diabetes, targeting β-cell dysfunction and disease progression. In **Table 1** the pharmacological agents that modulate ER Stress in diabetes with the target pathway involved are presented. Chaperone molecules like 4-phenylbutyric acid and tauroursodeoxycholic acid alleviate ER stress by enhancing protein folding capacity, improving insulin secretion and β-cell survival in diabetic models (63). Glucagon-like peptide-1 (GLP-1) receptor agonists reduce ER stress-induced apoptosis by activating unfolded protein response pathways, preserving β-cell function and protecting vascular endothelium by up-regulating the ER chaperone GRP78 and the anti-apoptotic protein JunB (64). In the same class of drugs, liraglutide showed protective effects on human umbilical vein endothelial cells from glucose-induced ER stress (65). The cardioprotective properties of SGLT2 inhibitors and GLP−1 agonists were independent of the glucose lowering effect (66). Kapadia et al. reported that part of this benefit stemmed from reduced ER stress based on experiments on primary human coronary artery endothelial cells (67). Exendin-4 attenuates palmitate-induced ER stress by targeting the IRE1α/XBP1 pathway and reducing pro-apoptotic signals (68, 69).

**Table 1:** Pharmacological agents modulating ER stress in diabetes.

Class/Examples	Mechanism of action	Therapeutic applications	UPR pathway/target	References
Chemical Chaperones - 4-Phenylbutyric acid (4-PBA) - Tauroursodeoxycholic acid (TUDCA)	Improve ER protein folding capacity, stabilize protein conformation, reduce misfolded protein accumulation, attenuate overactivated UPR	Improve insulin sensitivity, glucose tolerance, and β-cell function, studied in T2D, diabetic nephropathy, Wolfram syndrome	General enhancement of protein folding, reducing stress on all UPR branches (PERK, IRE1α, ATF6)	Ni et al. (63) Kusaczuk et al. (75)
Pathway-Specific Inhibitors - GSK2606414 (PERK inhibitor) - MKC-3946 (IRE1α inhibitor) - Salubrinal (eIF2α phosphatase inhibitor) - Trazodone (ATF4 inhibitor)	Selectively block maladaptive UPR signaling pathways (PERK, IRE1, eIF2α, ATF4) involved in β-cell apoptosis	Experimental reduction of ER stress–induced β-cell death *in vitro* and *in vivo*	Target specific UPR branches: PERK–eIF2α–ATF4, IRE1α pathways	Ni et al. (63)
Antidiabetic Drugs - GLP-1 receptor agonists - Dapagliflozin - Metformin	Reduce ER stress and inflammatory signaling, enhance β-cell insulin secretion	Clinically used for T2D, ER stress modulation may contribute to β-cell preservation	Indirectly reduces ER stress by modulating NF-κB and other stress pathways AMPK activation and suppression of lipotoxicity-related pathways such as CD36	Tsunekawa et al. (68) Kapadia et al. (67)
Glucocorticoid receptor ligand (GR) - Compound A (CpdA)	Improves the UPR and attenuates ER stress in a GR independent manner	Favors the survival and function of β-cells in NOD SCID diabetic mice	CpdA inhibits the cytokine−induced activation of ER stress and favors UPR pathways in rat β-cell line	Andone et al. (74)

Metformin exerts pleiotropic effects by attenuating ER stress and oxidative stress in β-cells and endothelial cells thereby protecting them from glucotoxicity and apoptosis (70). More specifically, it reduces the palmitate-induced expression of ER stress markers such as GRP78/BiP and protein disulfide isomerase, suppresses activation of JNK and IRS-1 serine phosphorylation, and downregulates the pro-apoptotic transcription factor CHOP, thereby preserving β−cell viability (70). Additionally, it inhibits the expression of Cluster determinant 36 (CD36), a hyperglycemia-induced fatty acid transporter, that increases free fatty acid uptake and reduces insulin secretion. It also reduces reactive oxygen species (ROS) production, attenuating the β-cell apoptosis (67, 70). In the vascular endothelium, metformin protects human coronary artery endothelial cells under hyperglycemic conditions by suppressing critical ER stress markers including phosphorylated IRE1α, PERK, ATF6, and GRP78 (15). These effects ameliorate endothelial dysfunction, a key factor in the development of diabetic microvascular and macrovascular complications. In cardiac tissue, metformin selectively modulates ER stress pathways, activating certain adaptive arms such as PERK while suppressing the pro-apoptotic pathways via ATF4–CHOP. This results in cardioprotective effects without promoting cell death (71). The well-known actions of metformin through AMP-activated protein kinase (AMPK) and PI3K signaling synergistically alleviate oxidative stress, normalize ER homeostasis, and reduce apoptosis (72). Metformin also exerts neuroprotective effects by suppressing ER stress-induced apoptosis in neurons (73). In summary, metformin exhibits significant ER stress-modulating properties across multiple tissues involved in diabetes pathophysiology.

The selective inhibitors of UPR signaling branches (e.g., PERK and IRE1α inhibitors) show potential to prevent ER stress–mediated β-cell apoptosis in preclinical studies (63).

From the above drugs, the chemical chaperones 4-phenylbutyric acid and tauroursodeoxycholic acid as well as the selective UPR pathway inhibitors are currently experimental and have not been approved for clinical use in pediatric populations. Metformin is currently approved for use in pediatric patients, specifically for the treatment of T2D in children aged 10 years and older. It is widely used to improve glycemic control by reducing hepatic glucose production and improving insulin sensitivity, with additional benefits including modulation of ER stress (70). From the GLP-1 receptor agonists liraglutide has been recently approved for use in pediatric patients with T2D, typically for those aged 10 years and above, to improve glycemic control.

**Figure 2:**
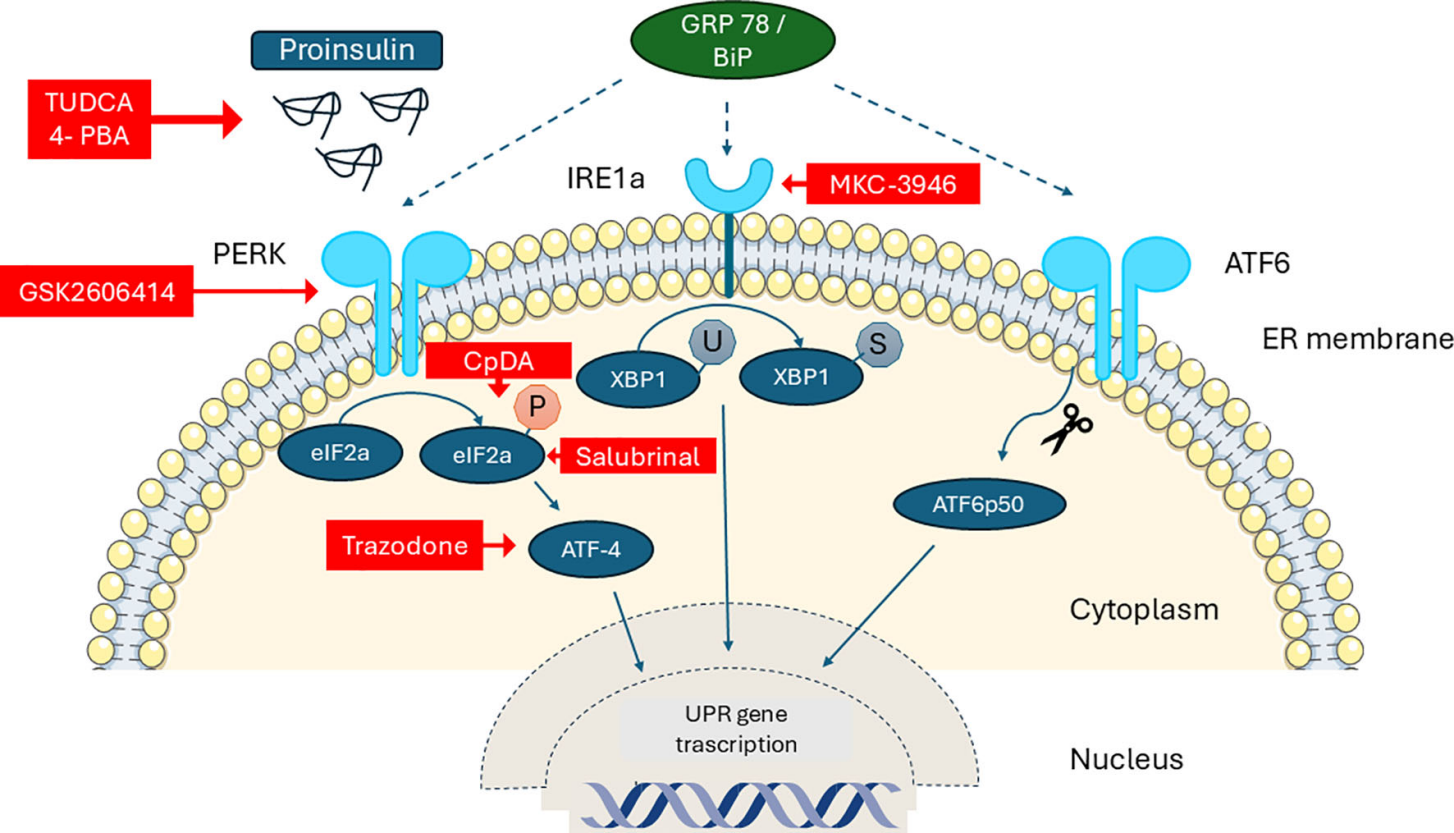
Schematic diagram of the unfolded protein response (UPR) in the β-cell and the sites of action of specific pharmacological modulators (red boxes). PERK: Protein Kinase R-like ER, IRE1a: Serine–threonine Protein Kinase–Endoribonuclease XBP-1: X-box Binding Protein-1, GRP78/BiP: 78 kDa Glucose-Regulated Protein/Binding immunoglobulin Protein, 4-PBA Phenylbutyric acid, TUDCA: Tauroursodeoxycholic acid, CpdA: Compound A.

Recently Andreone et al. reported that Compound A (CpdA), a small drug and selective glucocorticoid receptor (GR/NR3C1) ligand with inflammation-suppressing effects *in vivo*, modulates effector T cells, dendritic cells, and macrophages independently of the GR. They showed that, in NODscid mice given diabetogenic splenocytes from diabetic NOD mice, CpdA improves the UPR by reducing ER stress and supports the survival and function of β-cells. Pancreas histology showed less islet leukocyte infiltration (insulitis) and better preservation of insulin expression in CpdA-treated, normoglycemic mice compared to controls (74) (**Figure 2**).

## Non-pharmacological modulation of ER stress response in pediatric diabetes

Physical exercise has been shown to alleviate ER stress by activating the UPR (76). Exercise reduces the expression of glucose-regulated protein 78 (GRP78) and decreases the phosphorylation of stress-related proteins like IRE1α and eIF2α (77). Apart from the above direct effects on the ER function, exercise enhances the insulin sensitivity by increasing the post-receptor effects of insulin as evidenced by the increased phosphorylation of insulin receptor substrates and Akt activation (78). This effect has also been documented in the skeletal muscle of mice, where exercise reversed high-fat diet-induced ER stress and insulin resistance (79).

In addition, exercise activates the AMPK/PGC1α pathway, which participates in protective autophagy, thereby reducing ER stress and improving insulin resistance (79). Conclusively, exercise has a multidimensional action on modulating the ER response in terms of increased capacity and adaptability to stress. Of note is that in T2D patients exercise-induced regulation of stress response markers is not impaired compared to controls, suggesting that exercise remains beneficial in managing diabetes-related ER stress (80).

Studies have reported that treadmill running (81) and swimming (82), help lower the expression of ER stress markers like BiP, p-IRE1α, ATF6, p-PERK, p-eIF2α, ATF4, and cleaved ATF6 in the heart, liver, brain, and muscle tissues of diabetic animals. In addition, resistance training has been shown to significantly reduce the levels of ER stress-related genes BiP, XBP1, ATF4, and CHOP in mice (83).

Diets rich in antioxidants (such as sulforaphane, a compound found in broccoli), omega-3 fatty acids, and polyphenols can help reduce ER stress. For example, sulforaphane upregulates metabolic genes such as *AMPK* and *PPAR-α* while reducing inflammatory markers and ER stress signals like *CHOP*, improving liver health. Caloric restriction and intermittent fasting also show benefit by reducing oxidative and inflammatory triggers of ER stress (84). Additional lifestyle modifications like adequate sleep and avoidance of chronic psychological stress are beneficial for maintaining ER health within the context of diabetes.

Gene modulation and cell therapies are still experimental but show potential for treating conditions involving ER stress. Numerous studies have demonstrated that diverse range of long non-coding RNAs (lnc-RNAs) play a significant role in the regulation of ER stress pathways involved in various diseases associated with ER stress (85). For example, high-glucose treatment of ARPE-19 human adult retinal pigment epithelial cells results in a decrease of lncRNA GAS5 and an increase in ER stress by upregulating the expression levels of PERK, ATF4 and CCAAT/enhancer-binding protein homologous proteins. Therefore, manipulating lncRNA GAS5 levels by direct cellular reprogramming or gene editing could restore ER integrity and reduce diabetic retinopathy (86).

In conclusion, exercise exerts a systemic effect, impacting not only muscle but also the liver, adipose tissue, heart, and pancreas. Systemic exercise along with healthy nutrition and lifestyle integrates into an important axis for an optimal metabolic environment beneficial not only for the β-cells but all the active metabolic tissue of the human body.

## Discussion

While restoring euglycemia remains the primary goal in diabetes management, the manipulation of ER stress emerges as an additional therapeutic target in T1D and other forms of diabetes because of the accumulating evidence supporting that the ER stress is not merely a consequence, but a causative factor in diabetes pathogenesis. Persistent ER stress in pancreatic β-cells and other tissues, continues to drive inflammation, cellular senescence and finally apoptosis—even under good glycemic control—by perpetuating maladaptive unfolded protein-induced cycles of oxidative and inflammatory stress. Thus, it is increasingly clear that achieving normal blood glucose is not sufficient to halt β-cell dysfunction, insulin resistance, or diabetes-related organ damage. Methods to target ER stress alongside glycemic correction addresses core metabolic pathways that disrupt homeostasis, protects β-cell mass, reduces chronic complications, and offers a more comprehensive strategy for long-term metabolic health.

Within the context of pediatric diabetes, where medication or other experimental treatments are approached with great caution and typically extensive clinical trials are required, the first step is the adoption of systemic exercise in everyday life. Exercise plays an essential role, especially in children with both T1D or T2D who have achieved glycemic control. Successful modulation of ER stress through exercise following the diagnosis of either T1D or T2D may significantly retard disease progression by preserving residual β-cell mass and delaying β-cell apoptosis. It is also well established that exercise improves insulin resistance and decreases inflammation, thereby potentiating positively the ER stress reduction by maintaining a good homeostatic balance of the insulin signaling pathways. The non-pharmacological modulation of ER stress through systemic exercise is strongly based on the link between muscular health and ER stress with muscle having a pivotal role in mediating systemic cellular stress responses. Skeletal muscle, with its large tissue mass and highly active metabolic profile, serves as a critical site for glucose uptake and is intimately involved in regulating whole-body insulin sensitivity. Enhancing muscular health through exercise and nutrition except for the improved insulin action, the secretion of exerkines can exert distal effects on other organs, notably the pancreatic β-cells, by modulating their ER stress adaptation and reducing their loss. Therefore, strategies aimed at fostering muscular health not only provide local benefits but may also indirectly boost β-cell function. This translates to better clinical outcomes slowing the progression of diabetes-related complications.

The fact that different types of exercise affect distinct arms of the metabolic pathways that modulate ER stress (20), means that we should meticulously approach the physical activity programs to contain a mix of aerobic, anaerobic and resistance types of exercise. Nowadays, the role of exercise in alleviating ER stress has become more important, especially in the context of T1D. This is because glycemic control is often achieved using closed-loop insulin delivery systems, even in children who do not engage in physical activity. In such cases, higher insulin doses are frequently required to overcome insulin resistance, which can arise during adolescence and may be further aggravated by a sedentary lifestyle. Technological advances may achieve euglycemia yet mask an underlying metabolic state characterized by increased insulin resistance and elevated ER stress, which can affect residual β-cell mass and contribute long-term to both microvascular and macrovascular diabetic complications. Systematic training schedules can even be systematically evaluated in all other forms of diabetes where ER is demonstrated to have the central role in pathogenesis of β-cell insufficiency.

When discussing the pharmacological modulation of ER stress in pediatric diabetes, special attention should be given to metformin, because it is an old well-studied drug approved for use in children with insulin resistance. Metformin exhibits a significant re-purposing potential for alleviating ER stress. Beyond its primary anti-hyperglycemic action, metformin prevents glucotoxicity in pancreatic β-cells by suppressing oxidative and ER stress. These findings highlight metformin’s ability to modulate ER stress independently of its glucose-lowering effect, supporting the consideration of parallel use of metformin in T1D and T2D. Furthermore, metformin alleviates ER stress across multiple tissues beyond pancreas by acting on cardiac muscle, neurons, liver, and adipose tissue. In the last years, clinical studies supported the adjunctive use of metformin in people with T1D, particularly for those with insulin resistance, excessive insulin requirements, overweight or high cardiovascular risk (87, 88). With the addition of ER stress-modulation related data, it seems that metformin should acquire a new therapeutic role beyond glycemic control, in targeting the diabetes-related organ damage and complications, as a multifaceted regulator of cellular stress. It would be valuable to have systematic clinical trial data on metformin as an ER stress modulator in T1D and T2D, as well as in syndromic forms of diabetes where ER stress is known to play the causal role.

Focusing on T2D, recent studies have shown that defects in proinsulin/insulin processing and ER stress markers progressively worsen during the transition from normal glucose tolerance to impaired glucose tolerance, increased expression of ER stress-related genes and increased in β-cell workload (high insulin demand and insulin resistance) that consequently leads to loss of β-cell dysfunction ultimately leading to T2D (48). Given the fact that, in adolescents, impaired glucose tolerance state may progress faster to T2D than in adults (89) it would be of primary importance to implement early therapeutic strategies aimed at reducing ER stress help lessen β-cell workload and delay β-cell exhaustion and clinical progression to T2D.

Regarding the other ER stress modulators, a randomized, double-blind, placebo-controlled pilot study aimed to determine whether Tauroursodeoxycholic acid (TUDCA), an oral drug approved in Europe for gallstones and liver disease, can reduce ER stress and improve beta cell survival in patients with new-onset T1D has been registered (https://clinicaltrials.gov/study/NCT02218619). The main endpoint was the change in stimulated C-peptide secretion (a marker of insulin secretion and beta cell function) at 6-, 12-, and 18-months following treatment. Secondary outcomes included insulin use and HbA1c levels. The final results of this trial have not been published yet.

However, given the available data, there appears to be individual variability in susceptibility to ER stress and in UPR capacity, which could influence the effectiveness of both pharmacological and non-pharmacological ER-modulating therapies. Clinical trials are needed to evaluate ER modulators, while considering this interindividual difference in ER stress response and its modulation.

## Conclusion

Scientific data largely from translational and animal studies, highlight the causative role of ER stress in the development and progression of all forms of pediatric diabetes. The recently described beneficial effects of modulating ER stress on pancreatic β-cells and other tissues—through both pharmacological and non-pharmacological approaches—are opening a new research field on the optimal use of exercise and available drugs in the clinical context. This complements the traditional focus on achieving euglycemia with advanced insulin analogs and insulin delivery technologies, offering a broader strategy for improved diabetes management.
